# Supplementary material for: Urban pandemic response: Survey results describing the experiences from twenty-five cities during the COVID-19 pandemic
Source: PLOS Glob Public Health. 2022 Nov 29;2(11):e0000859. doi: 10.1371/journal.pgph.0000859 (PMC10021545; doi:10.1371/journal.pgph.0000859)
Supplement: S1 Text — (PDF) [file pgph.0000859.s001.pdf]

S1 Text. Survey Questionnaire.

Section I: Background and demographic information

1. **What is your full name?**

2. **What is your gender?**
   A. Male B. Female
   C. Other D. Prefer not to answer

3. **What is your preferred email address?**

4. **What city or urban area do you represent? (City, Country)**

5. **What is the name of your department, office, or agency?**

6. **What is your job title?**

7. **How many years have you been in this role?**
   A. Less than 1 year B. 1-4 years
   C. 5-9 years D. 10 years or more

8. **If your role and title changed as a result of the response to the COVID-19 pandemic, what was the name of your department/office and job title before the pandemic?**

Section II: COVID-19 Pandemic Response in Your City

9. **To the best of your ability, please list all national government ministries, agencies, departments, and offices involved in the response to the pandemic in your city.**

10. **To the best of your ability, please list all regional government (State/Province) ministries, agencies, departments, and offices involved in the response to the pandemic in your city.**

11. **To the best of your ability, please list all local government (County/District) agencies, departments, and offices involved in the response to the pandemic in your city.**

12. **To the best of your ability, please list all municipal/city government agencies, departments, and offices involved in the response to the pandemic in your city.**

13. **What structures (e.g., Emergency Operations Center) or mechanisms (e.g., scheduled calls/meetings) has your city government used to coordinate the local response with the national and/or regional response to the COVID-19 pandemic? Please describe briefly.**

14. **What role, if any, has data played in the decision-making processes of your city's government? Please describe briefly.**

15. **Which levels of government are responsible for the implementation of the following pandemic response activities in your city? Please select all levels of government that are responsible for implementing an activity in your city.**

Pandemic Response Activity	Level of Government				
	National	Regional	Local	Municipal	Not Applicable
Financing emergency public health response activities					
Mandating business closures					
Mandating curfews/lockdowns					
Mandating individual behavior changes (e.g., facemasks/social distancing)					
Mandating school closures					
Suspending routine public services (e.g. transit, sanitation, etc.)					
Conducting data analysis and surveillance activities					
Risk communication activities					
Maintaining essential health services					
Surging medical care to meet increased demand					
Coordinating with health care systems and organizations					
Coordinating with community-based and civil society organizations					
Providing diagnostic testing services					
Contact tracing activities					
Providing quarantine and isolation services					
Conducting immunization campaigns					

16. **Which level of government is accountable for the following pandemic response activities in your city? Please select the level of government that oversees an activity in your city.**

Pandemic Response Activity	Level of Government				
	National	Regional	Local	Municipal	Not Applicable
Financing emergency public health response activities					
Mandating business closures					
Mandating curfews/lockdowns					
Mandating individual behavior changes (e.g., facemasks/social distancing)					
Mandating school closures					
Suspending routine public services (e.g. transit, sanitation, etc.)					
Conducting data analysis and surveillance activities					
Risk communication activities					
Maintaining essential health services					
Surging medical care to meet increased demand					
Coordinating with health care systems and organizations					
Coordinating with community-based and civil society organizations					
Providing diagnostic testing services					
Contact tracing activities					
Providing quarantine and isolation services					
Conducting immunization campaigns					

17. **Which levels of government are consulted (i.e., provide information and with whom there is two-way communication) for the following pandemic response activities in your city? Please select all levels of government that are consulted for an activity in your city.**

Pandemic Response Activity	Level of Government				
	National	Regional	Local	Municipal	Not Applicable
Financing emergency public health response activities					
Mandating business closures					
Mandating curfews/lockdowns					
Mandating individual behavior changes (e.g., facemasks/social distancing)					
Mandating school closures					
Suspending routine public services (e.g. transit, sanitation, etc.)					
Conducting data analysis and surveillance activities					
Risk communication activities					
Maintaining essential health services					
Surging medical care to meet increased demand					
Coordinating with health care systems and organizations					
Coordinating with community-based and civil society organizations					
Providing diagnostic testing services					
Contact tracing activities					
Providing quarantine and isolation services					
Conducting immunization campaigns					

18. **Which levels of government are informed (i.e., informed of updates and with whom there is one-way communication) about the following pandemic response activities in your city? Please select all levels of government that are informed about an activity in your city.**

Pandemic Response Activity	Level of Government				
	National	Regional	Local	Municipal	Not Applicable
Financing emergency public health response activities					
Mandating business closures					
Mandating curfews/lockdowns					
Mandating individual behavior changes (e.g., facemasks/social distancing)					
Mandating school closures					
Suspending routine public services (e.g. transit, sanitation, etc.)					
Conducting data analysis and surveillance activities					
Risk communication activities					
Maintaining essential health services					
Surging medical care to meet increased demand					
Coordinating with health care systems and organizations					
Coordinating with community-based and civil society organizations					
Providing diagnostic testing services					
Contact tracing activities					
Providing quarantine and isolation services					
Conducting immunization campaigns					

19. When did your city government begin to implement the following actions to respond to the pandemic?

Response Activity	Time Action Was Taken by City Government				
	Before confirmation of first case in your country	After confirmation of first case in country but before first case in your city	After confirmation of first case in your city	City government has not implemented this activity	Not Applicable
Financing emergency public health response activities					
Mandating business closures					
Mandating curfews/lockdowns					
Mandating individual behavior changes (e.g., facemasks/social distancing)					
Mandating school closures					
Suspending routine public services (e.g. transit, sanitation, etc.)					
Conducting data analysis and surveillance activities					
Risk communication activities					
Maintaining essential health services					
Surging medical care to meet increased demand					
Coordinating with health care systems and organizations					
Coordinating with community-based, non-governmental organizations					
Providing diagnostic testing services					
Contact tracing activities					
Providing quarantine and isolation services					
Conducting immunization campaigns					

20. Did your city government take other action(s) to respond to the pandemic? Please describe briefly.

21. How challenging were the following response activities for your city in responding to the COVID-19 pandemic?

	Extremely challenging	Very challenging	Challenging	Slightly challenging	Not challenging at all	Not Applicable
Financing emergency public health response activities						
Mandating business closures						
Mandating curfews/lockdowns						
Mandating individual behavior changes (e.g., facemasks/social distancing)						
Mandating school closures						
Suspending routine public services (e.g. transit, sanitation, etc.)						
Risk communication activities						
Maintaining essential health services						
Surging medical care to meet increased demand						
Coordinating with health care systems and organizations						
Coordinating with community-based, non-governmental organizations						
Providing diagnostic testing services						
Contact tracing activities						
Providing quarantine and isolation services						
Conducting immunization campaigns						

22. **How challenging were the following risk communication activities for your city during the response to the COVID-19 pandemic?**

	Extremely challenging	Very challenging	Challenging	Slightly challenging	Not challenging at all	Not Applicable
Intergovernmental communications (communicating with higher levels of government)						
External communications with the media						
External communications with local businesses and organizations						
External communications with the general public						
Internal communications (within city government)						
Reaching high-risk populations with information						
Addressing misinformation and disinformation						

23. **What was the biggest challenge your city faced in pandemic response? Please explain briefly.**

24. **Did your city enjoy any successes in the response to the pandemic or conduct any response activities particularly well? Please describe briefly.**

25. **How helpful was the PHC grant funding useful for improving your city's response to the COVID-19 pandemic?**
    A. Extremely helpful
    B. Very helpful
    C. Helpful
    D. Slightly helpful
    E. Not helpful at all

26. **In the future, what models should PHC consider to better support the response to epidemics or pandemics in cities? Please select all that apply.**
    A. Direct financial assistance
    B. Material assistance (i.e., equipment)
    C. Personnel assistance
    D. Technical assistance
    E. Other (please describe)

Section III: Future Efforts in Your City

27. **Following COVID-19, do you think your city government will be more engaged in pandemic or epidemic preparedness?**
    A. Yes
    B. No
    C. I don't know/I am unsure

28. **What type of support does your city need from external partners to improve future epidemic preparedness? Please select all that apply.**
    A. Direct financial assistance
    B. Material assistance (i.e., equipment)
    C. Personnel assistance
    D. Technical assistance
    E. Other (please describe)

29. **What are the three most important things to improve future pandemic preparedness and response in your city? Please describe briefly.**

30. **Do cities offer any comparative advantages in epidemic or pandemic preparedness and response over other levels of government? Please describe briefly.**
