# Supplementary material for: Urban pandemic response: Survey results describing the experiences from twenty-five cities during the COVID-19 pandemic
Source: PLOS Glob Public Health. 2022 Nov 29;2(11):e0000859. doi: 10.1371/journal.pgph.0000859 (PMC10021545; doi:10.1371/journal.pgph.0000859)
Supplement: S1 Data — (PDF) [file pgph.0000859.s002.pdf]

S1 Data. Urban Pandemic Response RACI Matrices.

Legend		
**Governance Level**	National (N)	*National government*
	Subnational (S)	*State, provincial, district, or county governments*
	Local (L)	*City or municipal governments*
**RACI Roles**	Responsible (R)	*The level of government that implemented the work required to complete an activity*
	Accountable (A)	*The level of government that oversaw the correct and thorough completion of an activity*
	Consulted (C)	*Levels of government engaging in two-way communication to provide information necessary for the completion of an activity*
	Informed (I)	*Level of government that was updated regarding a given activity (i.e., one-way communication)*

	**Activity**	**N**	**S**	**L**
**Accra, Ghana**	Financing emergency public health response activities	RA	RI	RI
	Mandating business closures	A	RC	I
	Mandating curfews/lockdowns	RA	I	I
	Mandating individual behavior changes (e.g., facemasks/social distancing)	RA	RC	RC
	Mandating school closures	RA	C	I
	Suspending routine public services (e.g. transit, sanitation, etc.)	RA	C	C
	Conducting data analysis and surveillance activities	RA	RC	R
	Risk communication activities	RA	RC	R
	Maintaining essential health services	RA	RC	RC
	Surging medical care to meet increased demand	RA	RC	RC
	Coordinating with health care systems and organizations	RC	RA	RC
	Coordinating with community-based and civil society organizations	RC	RA	RC
	Providing diagnostic testing services	RA	C	C
	Contact tracing activities	C	RA	C
	Providing quarantine and isolation services	RA	RC	C
	Conducting immunization campaigns	RI	RA	RC

Addis Ababa, Ethiopia	Activity	N	S	L
	Financing emergency public health response activities	RA	R	
	Mandating business closures	C	R	RA
	Mandating curfews/lockdowns	RA	R	RC
	Mandating individual behavior changes (e.g., facemasks/social distancing)	RC	RC	A
	Mandating school closures	RA	R	C
	Suspending routine public services (e.g. transit, sanitation, etc.)	RA	R	
	Conducting data analysis and surveillance activities	RC	RC	RA
	Risk communication activities	RC	RC	RA
	Maintaining essential health services	RC	RC	A
	Surging medical care to meet increased demand	RC	R	RA
	Coordinating with health care systems and organizations	RA	RC	C
	Coordinating with community-based and civil society organizations	RC	RC	RA
	Providing diagnostic testing services	RA	RC	RC
	Contact tracing activities	RC	RC	RA
	Providing quarantine and isolation services	RC	RC	A
	Conducting immunization campaigns	RC	RC	A

Amman, Jordan	Activity	N	S	L
	Financing emergency public health response activities	RA		
	Mandating business closures	RA		
	Mandating curfews/lockdowns	RA		
	Mandating individual behavior changes (e.g., facemasks/social distancing)	RA		
	Mandating school closures	RA		
	Suspending routine public services (e.g. transit, sanitation, etc.)	RA		
	Conducting data analysis and surveillance activities	RA		
	Risk communication activities	RA		R
	Maintaining essential health services	RA		
	Surging medical care to meet increased demand	RA		
	Coordinating with health care systems and organizations	RA		
	Coordinating with community-based and civil society organizations	RA		
	Providing diagnostic testing services	RA		
	Contact tracing activities	RA		
	Providing quarantine and isolation services	RA		
	Conducting immunization campaigns	RA		

	Activity	N	S	L
**Athens, Greece**	Financing emergency public health response activities	RA		R
	Mandating business closures	RA		
	Mandating curfews/lockdowns	RA		
	Mandating individual behavior changes (e.g., facemasks/social distancing)	RA		
	Mandating school closures	RA		
	Suspending routine public services (e.g. transit, sanitation, etc.)	RA		
	Conducting data analysis and surveillance activities	RA		
	Risk communication activities	RA		
	Maintaining essential health services	RA		R
	Surging medical care to meet increased demand	RA		R
	Coordinating with health care systems and organizations	RA		
	Coordinating with community-based and civil society organizations			RA
	Providing diagnostic testing services	RA	R	R
	Contact tracing activities	RA		
	Providing quarantine and isolation services	RA		
	Conducting immunization campaigns	RA		

	Activity	N	S	L
**Bandung, Indonesia**	Financing emergency public health response activities	RA	RC	RC
	Mandating business closures	RC	RA	RC
	Mandating curfews/lockdowns	RA	RC	RC
	Mandating individual behavior changes (e.g., facemasks/social distancing)	RC	RC	RA
	Mandating school closures	RC	RA	RC
	Suspending routine public services (e.g. transit, sanitation, etc.)	RA	RC	RC
	Conducting data analysis and surveillance activities	C	RC	RA
	Risk communication activities	RC	RC	RA
	Maintaining essential health services	RC	RC	RA
	Surging medical care to meet increased demand	RC	RA	RC
	Coordinating with health care systems and organizations	R	RA	RC
	Coordinating with community-based and civil society organizations	C	RC	RA
	Providing diagnostic testing services	RC	RC	RA
	Contact tracing activities	C	RC	RA
	Providing quarantine and isolation services	RC	RC	RA
	Conducting immunization campaigns	RC	RC	RA

Bangkok, Thailand	Activity	N	S	L
	Financing emergency public health response activities	RA	RC	I
	Mandating business closures	RA	RC	I
	Mandating curfews/lockdowns	RA	RC	I
	Mandating individual behavior changes (e.g., facemasks/social distancing)	RA	RC	I
	Mandating school closures	RA	RC	I
	Suspending routine public services (e.g. transit, sanitation, etc.)	RA	RC	I
	Conducting data analysis and surveillance activities	RA	C	I
	Risk communication activities	RA	RC	I
	Maintaining essential health services	RA	RC	I
	Surging medical care to meet increased demand	RA	RC	I
	Coordinating with health care systems and organizations	RA	RC	I
	Coordinating with community-based and civil society organizations	RA	RC	I
	Providing diagnostic testing services	RA	RC	I
	Contact tracing activities	RA	RC	I
	Providing quarantine and isolation services	RA	RC	I
	Conducting immunization campaigns	RA	RC	I

Barcelona, Spain	Activity	N	S	L
	Financing emergency public health response activities		RA	C
	Mandating business closures		RA	C
	Mandating curfews/lockdowns	RI	RA	C
	Mandating individual behavior changes (e.g., facemasks/social distancing)	RI	RA	C
	Mandating school closures		RC	A
	Suspending routine public services (e.g. transit, sanitation, etc.)	R	A	C
	Conducting data analysis and surveillance activities	R	RC	RA
	Risk communication activities	R	RC	RA
	Maintaining essential health services	RI	RA	C
	Surging medical care to meet increased demand		RA	C
	Coordinating with health care systems and organizations	R	RA	RC
	Coordinating with community-based and civil society organizations		RC	RA
	Providing diagnostic testing services	C	RA	I
	Contact tracing activities		C	RA
	Providing quarantine and isolation services		C	RA
	Conducting immunization campaigns	RC	RA	RI

Bengaluru, India

Activity	N	S	L
Financing emergency public health response activities		RA	R
Mandating business closures		RC	RA
Mandating curfews/lockdowns		RC	RA
Mandating individual behavior changes (e.g., facemasks/social distancing)		RC	RA
Mandating school closures		RC	A
Suspending routine public services (e.g. transit, sanitation, etc.)		RC	A
Conducting data analysis and surveillance activities		RC	RA
Risk communication activities		RC	RA
Maintaining essential health services		RC	RA
Surging medical care to meet increased demand		RA	RC
Coordinating with health care systems and organizations		RC	RA
Coordinating with community-based and civil society organizations		RC	RA
Providing diagnostic testing services		RC	RA
Contact tracing activities			RA
Providing quarantine and isolation services			RA
Conducting immunization campaigns		RC	RA

Buenos Aires, Argentina

Activity	N	S	L
Financing emergency public health response activities	RC	RA	
Mandating business closures	RC	A	
Mandating curfews/lockdowns	RC	A	
Mandating individual behavior changes (e.g., facemasks/social distancing)	RC	RA	
Mandating school closures	RC	RA	
Suspending routine public services (e.g. transit, sanitation, etc.)	RC	RA	
Conducting data analysis and surveillance activities		RA	
Risk communication activities		RA	
Maintaining essential health services	I	RA	
Surging medical care to meet increased demand	I	RA	
Coordinating with health care systems and organizations	RI	RA	
Coordinating with community-based and civil society organizations		RA	
Providing diagnostic testing services		RA	
Contact tracing activities		RA	
Providing quarantine and isolation services		RA	
Conducting immunization campaigns	I	RA	

Cali, Colombia

Activity	N	S	L
Financing emergency public health response activities	RA	RC	
Mandating business closures	RI		RA
Mandating curfews/lockdowns	I		RA
Mandating individual behavior changes (e.g., facemasks/social distancing)	I		RA
Mandating school closures	RC	I	RA
Suspending routine public services (e.g. transit, sanitation, etc.)	I		RA
Conducting data analysis and surveillance activities	RC	RI	RA
Risk communication activities		RC	RA
Maintaining essential health services		RC	RA
Surging medical care to meet increased demand		RC	RA
Coordinating with health care systems and organizations		RC	RA
Coordinating with community-based and civil society organizations		RC	RA
Providing diagnostic testing services			RA
Contact tracing activities			RA
Providing quarantine and isolation services		R	RA
Conducting immunization campaigns	C	RC	RA

Colombo, Sri Lanka

Activity	N	S	L
Financing emergency public health response activities	C		RA
Mandating business closures	RA		I
Mandating curfews/lockdowns	RA		I
Mandating individual behavior changes (e.g., facemasks/social distancing)	RA		I
Mandating school closures	RA		I
Suspending routine public services (e.g. transit, sanitation, etc.)	A		RI
Conducting data analysis and surveillance activities	RA		R
Risk communication activities	RA		RI
Maintaining essential health services	RA		
Surging medical care to meet increased demand	RA		
Coordinating with health care systems and organizations	RA		R
Coordinating with community-based and civil society organizations	RA		R
Providing diagnostic testing services	RA		
Contact tracing activities	RA		R
Providing quarantine and isolation services	RA		R
Conducting immunization campaigns	RA		R

Guadalajara, Mexico	Activity	N	S	L
	Financing emergency public health response activities	RA	RC	RC
	Mandating business closures	RC	RA	RI
	Mandating curfews/lockdowns	RC	RA	R
	Mandating individual behavior changes (e.g., facemasks/social distancing)	RC	RC	RA
	Mandating school closures	RC	RC	RA
	Suspending routine public services (e.g. transit, sanitation, etc.)		I	RA
	Conducting data analysis and surveillance activities	RC	RA	RC
	Risk communication activities	RC	RA	RC
	Maintaining essential health services	RC	RC	RA
	Surging medical care to meet increased demand	RC	RA	RC
	Coordinating with health care systems and organizations	RC	RA	RC
	Coordinating with community-based and civil society organizations			RA
	Providing diagnostic testing services	RC	RA	RC
	Contact tracing activities		RA	RC
	Providing quarantine and isolation services		I	RA
	Conducting immunization campaigns	RA	RI	RI

Kampala, Uganda	Activity	N	S	L
	Financing emergency public health response activities	C	C	RA
	Mandating business closures		C	RA
	Mandating curfews/lockdowns	RC	RA	RC
	Mandating individual behavior changes (e.g., facemasks/social distancing)	C	C	RA
	Mandating school closures	RC	RC	RA
	Suspending routine public services (e.g. transit, sanitation, etc.)	RC	RC	RA
	Conducting data analysis and surveillance activities	RC		RA
	Risk communication activities	RC		RA
	Maintaining essential health services	C		RA
	Surging medical care to meet increased demand	RC	R	RA
	Coordinating with health care systems and organizations	RC		RA
	Coordinating with community-based and civil society organizations	RC	I	RA
	Providing diagnostic testing services	C		RA
	Contact tracing activities	C		RA
	Providing quarantine and isolation services	RA		RC
	Conducting immunization campaigns			RA

Kigali, Rwanda	Activity	N	S	L
	Financing emergency public health response activities	RA	I	RC
	Mandating business closures	RI	RC	RA
	Mandating curfews/lockdowns	RI	RC	RA
	Mandating individual behavior changes (e.g., facemasks/social distancing)	I	RC	RA
	Mandating school closures	RA	I	I
	Suspending routine public services (e.g. transit, sanitation, etc.)	RI	RI	RA
	Conducting data analysis and surveillance activities	RA	RI	RI
	Risk communication activities	I	RI	RA
	Maintaining essential health services	I	RI	RA
	Surging medical care to meet increased demand	I	RI	RA
	Coordinating with health care systems and organizations	RC	RI	RA
	Coordinating with community-based and civil society organizations	I	RI	RA
	Providing diagnostic testing services	RA	I	I
	Contact tracing activities	I	RI	RA
	Providing quarantine and isolation services	I	RI	RA
	Conducting immunization campaigns	I	RI	RA

Kumasi, Ghana	Activity	N	S	L
	Financing emergency public health response activities	RA	I	RC
	Mandating business closures	RA	C	RC
	Mandating curfews/lockdowns	RA	I	I
	Mandating individual behavior changes (e.g., facemasks/social distancing)	RA	I	C
	Mandating school closures	RA	I	C
	Suspending routine public services (e.g. transit, sanitation, etc.)	RA	I	RC
	Conducting data analysis and surveillance activities	RC	R	RA
	Risk communication activities	RC	I	RA
	Maintaining essential health services	RA	I	R
	Surging medical care to meet increased demand	RA	I	R
	Coordinating with health care systems and organizations	RA	R	RC
	Coordinating with community-based and civil society organizations	RC	C	RA
	Providing diagnostic testing services	RA	I	I
	Contact tracing activities	RA	I	RC
	Providing quarantine and isolation services	RA	I	C
	Conducting immunization campaigns	RA	I	RC

Lima, Peru	Activity	N	S	L
	Financing emergency public health response activities	RA	C	RC
	Mandating business closures	RA	C	I
	Mandating curfews/lockdowns	RA	C	I
	Mandating individual behavior changes (e.g., facemasks/social distancing)	RC	RC	RA
	Mandating school closures	RA	C	I
	Suspending routine public services (e.g. transit, sanitation, etc.)	RA	C	RC
	Conducting data analysis and surveillance activities	RA	C	R
	Risk communication activities	RA	RC	R
	Maintaining essential health services	RA	RC	RC
	Surging medical care to meet increased demand	RA	RC	RC
	Coordinating with health care systems and organizations	RA	C	C
	Coordinating with community-based and civil society organizations	RA	RC	RC
	Providing diagnostic testing services	RA	RC	RC
	Contact tracing activities	RA	RC	RC
	Providing quarantine and isolation services	RA	RC	RC
	Conducting immunization campaigns	RA	RC	RC

London, United Kingdom	Activity	N	S	L
	Financing emergency public health response activities	RA	RC	C
	Mandating business closures	A	RC	C
	Mandating curfews/lockdowns	A	RC	C
	Mandating individual behavior changes (e.g., facemasks/social distancing)	RA	RC	RC
	Mandating school closures	RA	RC	I
	Suspending routine public services (e.g. transit, sanitation, etc.)	A	RC	RC
	Conducting data analysis and surveillance activities	RA	RC	R
	Risk communication activities	RA	RC	RC
	Maintaining essential health services		C	C
	Surging medical care to meet increased demand		C	C
	Coordinating with health care systems and organizations		RC	RA
	Coordinating with community-based and civil society organizations	C	RC	RA
	Providing diagnostic testing services	A	RC	C
	Contact tracing activities	A	RC	C
	Providing quarantine and isolation services	A	RC	C
	Conducting immunization campaigns	C	C	C

	Activity	N	S	L
**Lusaka, Zambia**	Financing emergency public health response activities	RC	C	A
	Mandating business closures	RI	RC	RA
	Mandating curfews/lockdowns	RI	C	A
	Mandating individual behavior changes (e.g., facemasks/social distancing)	R	RC	RA
	Mandating school closures	RC	C	A
	Suspending routine public services (e.g. transit, sanitation, etc.)	RC	C	A
	Conducting data analysis and surveillance activities	RI	RC	A
	Risk communication activities	RC	RC	RA
	Maintaining essential health services	RA	RC	RC
	Surging medical care to meet increased demand	RC	A	
	Coordinating with health care systems and organizations	RC	RA	RC
	Coordinating with community-based and civil society organizations		C	RA
	Providing diagnostic testing services	RC	RC	A
	Contact tracing activities		RC	A
	Providing quarantine and isolation services	RA	RC	RC
	Conducting immunization campaigns	RC	RC	RA

	Activity	N	S	L
**Medellín, Colombia**	Financing emergency public health response activities	RC	RC	RA
	Mandating business closures	RC	RC	RA
	Mandating curfews/lockdowns	RC	RC	RA
	Mandating individual behavior changes (e.g., facemasks/social distancing)	RC	RC	RA
	Mandating school closures	RC	RC	RA
	Suspending routine public services (e.g. transit, sanitation, etc.)	RC	RC	RA
	Conducting data analysis and surveillance activities	RC	RC	RA
	Risk communication activities	RC	RC	RA
	Maintaining essential health services	RC	RC	RA
	Surging medical care to meet increased demand	RC	RC	RA
	Coordinating with health care systems and organizations	RC	RC	RA
	Coordinating with community-based and civil society organizations	C	RC	RA
	Providing diagnostic testing services	RC	RC	RA
	Contact tracing activities	RC	RC	RA
	Providing quarantine and isolation services	RC	RC	RA
	Conducting immunization campaigns	RC	RC	RA

	Activity	N	S	L
Ouagadougou, Burkina Faso	Financing emergency public health response activities	RA	R	RC
	Mandating business closures	RC		RA
	Mandating curfews/lockdowns	RA	R	C
	Mandating individual behavior changes (e.g., facemasks/social distancing)	RC		RA
	Mandating school closures	RA		RC
	Suspending routine public services (e.g. transit, sanitation, etc.)	RA		RC
	Conducting data analysis and surveillance activities	RA		I
	Risk communication activities	RC		RA
	Maintaining essential health services	RA		C
	Surging medical care to meet increased demand	RA		C
	Coordinating with health care systems and organizations	RA		I
	Coordinating with community-based and civil society organizations	C		RA
	Providing diagnostic testing services	RA		
	Contact tracing activities	RA		
	Providing quarantine and isolation services	RA		
	Conducting immunization campaigns	RA		

	Activity	N	S	L
Rio de Janeiro, Brazil	Financing emergency public health response activities	RA	RC	RC
	Mandating business closures	RC	RC	RA
	Mandating curfews/lockdowns	RC	RC	RA
	Mandating individual behavior changes (e.g., facemasks/social distancing)	RC	RC	RA
	Mandating school closures	RC	RC	RA
	Suspending routine public services (e.g. transit, sanitation, etc.)	RC	RC	RA
	Conducting data analysis and surveillance activities	RC	RC	RA
	Risk communication activities	RC	RC	RA
	Maintaining essential health services	RA	RC	RC
	Surging medical care to meet increased demand	RA	RC	RC
	Coordinating with health care systems and organizations	RA	RC	RC
	Coordinating with community-based and civil society organizations	RC	RC	RA
	Providing diagnostic testing services	RC	RC	RA
	Contact tracing activities	RC	RC	RA
	Providing quarantine and isolation services	RC	RC	RA
	Conducting immunization campaigns	RA	RC	RC

Santiago, Chile	Activity	N	S	L
	Financing emergency public health response activities	RC	RA	I
	Mandating business closures	RC	RA	I
	Mandating curfews/lockdowns	RC	RA	I
	Mandating individual behavior changes (e.g., facemasks/social distancing)	RC	RA	I
	Mandating school closures	RC	RA	I
	Suspending routine public services (e.g. transit, sanitation, etc.)	RC	RA	I
	Conducting data analysis and surveillance activities	RC	RA	I
	Risk communication activities	RC	RA	RI
	Maintaining essential health services	RC	RA	RI
	Surging medical care to meet increased demand	RC	RA	RI
	Coordinating with health care systems and organizations	RC	RA	RI
	Coordinating with community-based and civil society organizations	C	RA	RI
	Providing diagnostic testing services	C	RA	RI
	Contact tracing activities	RC	RA	RI
	Providing quarantine and isolation services	C	RA	RI
	Conducting immunization campaigns	RC	RA	RI

Santo Domingo, Dominican Republic	Activity	N	S	L
	Financing emergency public health response activities	RA	RI	RI
	Mandating business closures	RA	I	I
	Mandating curfews/lockdowns	RA	I	I
	Mandating individual behavior changes (e.g., facemasks/social distancing)	RA	I	I
	Mandating school closures	RA	I	I
	Suspending routine public services (e.g. transit, sanitation, etc.)	RA	I	I
	Conducting data analysis and surveillance activities	RA	RI	I
	Risk communication activities	RA	RI	RI
	Maintaining essential health services	RA	RI	I
	Surging medical care to meet increased demand	RA	I	I
	Coordinating with health care systems and organizations	RA	I	I
	Coordinating with community-based and civil society organizations	RA	RI	RI
	Providing diagnostic testing services	RA	RI	I
	Contact tracing activities	RA	RI	I
	Providing quarantine and isolation services	RA	I	I
	Conducting immunization campaigns	RA	RI	I

	Activity	N	S	L
**Vancouver, Canada**	Financing emergency public health response activities	R	RA	RC
	Mandating business closures		RA	I
	Mandating curfews/lockdowns		RA	I
	Mandating individual behavior changes (e.g., facemasks/social distancing)		RA	I
	Mandating school closures		RA	I
	Suspending routine public services (e.g. transit, sanitation, etc.)		RA	RI
	Conducting data analysis and surveillance activities	R	RA	RI
	Risk communication activities	R	RA	RC
	Maintaining essential health services	C	RA	
	Surging medical care to meet increased demand	C	RA	
	Coordinating with health care systems and organizations		RA	R
	Coordinating with community-based and civil society organizations		RA	RC
	Providing diagnostic testing services		RA	I
	Contact tracing activities		RA	
	Providing quarantine and isolation services	R	RA	
	Conducting immunization campaigns		RA	I

	Activity	N	S	L
**Yangon, Myanmar**	Financing emergency public health response activities	RC	RA	R
	Mandating business closures	RA	RC	R
	Mandating curfews/lockdowns	RA	RC	
	Mandating individual behavior changes (e.g., facemasks/social distancing)	RA	C	
	Mandating school closures	RA	C	
	Suspending routine public services (e.g. transit, sanitation, etc.)	RA	C	
	Conducting data analysis and surveillance activities	RA	RC	R
	Risk communication activities	RA	RC	R
	Maintaining essential health services	RA	RC	R
	Surging medical care to meet increased demand	RA	RC	R
	Coordinating with health care systems and organizations	RA	RC	R
	Coordinating with community-based and civil society organizations	RA	RC	R
	Providing diagnostic testing services	RA	RC	R
	Contact tracing activities	RA	RC	R
	Providing quarantine and isolation services	RA	RC	R
	Conducting immunization campaigns	RA	RC	
