# Supplementary material for: Urban pandemic response: Survey results describing the experiences from twenty-five cities during the COVID-19 pandemic
Source: PLOS Glob Public Health. 2022 Nov 29;2(11):e0000859. doi: 10.1371/journal.pgph.0000859 (PMC10021545; doi:10.1371/journal.pgph.0000859)
Supplement: S2 Data — (PDF) [file pgph.0000859.s003.pdf]

S2 Data. Survey Responses.

Variable	Description	Values
city	City name	NA
time1	Time financing emergency public health response activities began	1 = Before confirmed in country | 2 = After confirmed in country, but before city | 3 = After confirmed in city | 4 = Not implemented | 5 = Not applicable
time2	Time mandating business closures began	1 = Before confirmed in country | 2 = After confirmed in country, but before city | 3 = After confirmed in city | 4 = Not implemented | 5 = Not applicable
time3	Time mandating curfews/lock-downs began	1 = Before confirmed in country | 2 = After confirmed in country, but before city | 3 = After confirmed in city | 4 = Not implemented | 5 = Not applicable
time4	Time mandating individual behavior changes	1 = Before confirmed in country | 2 = After confirmed in country, but before city | 3 = After confirmed in city | 4 = Not implemented | 5 = Not applicable
time5	Time mandating school closures began	1 = Before confirmed in country | 2 = After confirmed in country, but before city | 3 = After confirmed in city | 4 = Not implemented | 5 = Not applicable
time6	Time suspending routine public services	1 = Before confirmed in country | 2 = After confirmed in country, but before city | 3 = After confirmed in city | 4 = Not implemented | 5 = Not applicable
time7	Time conducting data analysis and surveillance activities began	1 = Before confirmed in country | 2 = After confirmed in country, but before city | 3 = After confirmed in city | 4 = Not implemented | 5 = Not applicable
time8	Time risk communication activities began	1 = Before confirmed in country | 2 = After confirmed in country, but before city | 3 = After confirmed in city | 4 = Not implemented | 5 = Not applicable
time9	Time maintaining essential health services began	1 = Before confirmed in country | 2 = After confirmed in country, but before city | 3 = After confirmed in city | 4 = Not implemented | 5 = Not applicable
time10	Time surging medical care to meet increased demand began	1 = Before confirmed in country | 2 = After confirmed in country, but before city | 3 = After confirmed in city | 4 = Not implemented | 5 = Not applicable
time11	Time coordinating with health care systems and organizations began	1 = Before confirmed in country | 2 = After confirmed in country, but before city | 3 = After confirmed in city | 4 = Not implemented | 5 = Not applicable
time12	Time coordinating with CBOs and CSOs	1 = Before confirmed in country | 2 = After confirmed in country, but before city | 3 = After confirmed in city | 4 = Not implemented | 5 = Not applicable
time13	Time providing diagnostic testing services began	1 = Before confirmed in country | 2 = After confirmed in country, but before city | 3 = After confirmed in city | 4 = Not implemented | 5 = Not applicable
time14	Time contact tracing activities began	1 = Before confirmed in country | 2 = After confirmed in country, but before city | 3 = After confirmed in city | 4 = Not implemented | 5 = Not applicable
time15	Time providing quarantine and isolation services began	1 = Before confirmed in country | 2 = After confirmed in country, but before city | 3 = After confirmed in city | 4 = Not implemented | 5 = Not applicable
time16	Time conducting immunization campaigns began	1 = Before confirmed in country | 2 = After confirmed in country, but before city | 3 = After confirmed in city | 4 = Not implemented | 5 = Not applicable
chal1	Challenge of financing emergency public health response activities	1 = Extremely challenging | 2 = Very challenging | 3 = Challenging | 4 = Slightly challenging | 5 = Not challenging at all | 6 = Not applicable
chal2	Challenge of mandating business closures	1 = Extremely challenging | 2 = Very challenging | 3 = Challenging | 4 = Slightly challenging | 5 = Not challenging at all | 6 = Not applicable
chal3	Challenge of mandating curfews/lock-downs	1 = Extremely challenging | 2 = Very challenging | 3 = Challenging | 4 = Slightly challenging | 5 = Not challenging at all | 6 = Not applicable
chal4	Challenge of mandating individual behavior changes	1 = Extremely challenging | 2 = Very challenging | 3 = Challenging | 4 = Slightly challenging | 5 = Not challenging at all | 6 = Not applicable
chal5	Challenge of mandating school closures	1 = Extremely challenging | 2 = Very challenging | 3 = Challenging | 4 = Slightly challenging | 5 = Not challenging at all | 6 = Not applicable

chal6	Challenge of suspending routine public services	1 = Extremely challenging | 2 = Very challenging | 3 = Challenging | 4 = Slightly challenging | 5 = Not challenging at all | 6 = Not applicable
chal7	Challenge of conducting data analysis and surveillance activities	1 = Extremely challenging | 2 = Very challenging | 3 = Challenging | 4 = Slightly challenging | 5 = Not challenging at all | 6 = Not applicable
chal8	Challenge of risk communication activities	1 = Extremely challenging | 2 = Very challenging | 3 = Challenging | 4 = Slightly challenging | 5 = Not challenging at all | 6 = Not applicable
chal9	Challenge of maintaining essential health services	1 = Extremely challenging | 2 = Very challenging | 3 = Challenging | 4 = Slightly challenging | 5 = Not challenging at all | 6 = Not applicable
chal10	Challenge of surging medical care to meet increased demand	1 = Extremely challenging | 2 = Very challenging | 3 = Challenging | 4 = Slightly challenging | 5 = Not challenging at all | 6 = Not applicable
chal11	Challenge of coordinating with health care systems and organizations	1 = Extremely challenging | 2 = Very challenging | 3 = Challenging | 4 = Slightly challenging | 5 = Not challenging at all | 6 = Not applicable
chal12	Challenge of coordinating with CBOs and CSOs	1 = Extremely challenging | 2 = Very challenging | 3 = Challenging | 4 = Slightly challenging | 5 = Not challenging at all | 6 = Not applicable
chal13	Challenge of providing diagnostic testing services	1 = Extremely challenging | 2 = Very challenging | 3 = Challenging | 4 = Slightly challenging | 5 = Not challenging at all | 6 = Not applicable
chal14	Challenge of contact tracing activities	1 = Extremely challenging | 2 = Very challenging | 3 = Challenging | 4 = Slightly challenging | 5 = Not challenging at all | 6 = Not applicable
chal15	Challenge of providing quarantine and isolation services	1 = Extremely challenging | 2 = Very challenging | 3 = Challenging | 4 = Slightly challenging | 5 = Not challenging at all | 6 = Not applicable
chal16	Challenge of conducting immunization campaigns	1 = Extremely challenging | 2 = Very challenging | 3 = Challenging | 4 = Slightly challenging | 5 = Not challenging at all | 6 = Not applicable
cchal1	Challenge of intergovernmental communications	1 = Extremely challenging | 2 = Very challenging | 3 = Challenging | 4 = Slightly challenging | 5 = Not challenging at all | 6 = Not applicable
cchal2	Challenge of external communications - media	1 = Extremely challenging | 2 = Very challenging | 3 = Challenging | 4 = Slightly challenging | 5 = Not challenging at all | 6 = Not applicable
cchal3	Challenge of external communications - local businesses and organizations	1 = Extremely challenging | 2 = Very challenging | 3 = Challenging | 4 = Slightly challenging | 5 = Not challenging at all | 6 = Not applicable
cchal4	Challenge of external communications - general public	1 = Extremely challenging | 2 = Very challenging | 3 = Challenging | 4 = Slightly challenging | 5 = Not challenging at all | 6 = Not applicable
cchal5	Challenge of internal communications	1 = Extremely challenging | 2 = Very challenging | 3 = Challenging | 4 = Slightly challenging | 5 = Not challenging at all | 6 = Not applicable
cchal6	Challenge of reaching high-risk populations with information	1 = Extremely challenging | 2 = Very challenging | 3 = Challenging | 4 = Slightly challenging | 5 = Not challenging at all | 6 = Not applicable
cchal7	Challenge of addressing misinformation and disinformation	1 = Extremely challenging | 2 = Very challenging | 3 = Challenging | 4 = Slightly challenging | 5 = Not challenging at all | 6 = Not applicable
supp1	Open to direct financial support in the future	1 = Yes | 0 = No
supp2	Open to material assistance in the future	1 = Yes | 0 = No
supp3	Open to personnel assistance in the future	1 = Yes | 0 = No
supp4	Open to technical assistance in the future	1 = Yes | 0 = No
involve	Anticipated future engagement in pandemic preparedness	2 = More involved | 1 = Same or less involved | 0 = Unsure
theme1	Improvement theme discussed and/or referenced	1 = Coordination | 2 = Health systems strengthening | 3 = Human resources | 4 = Preparedness | 5 = Resourcing | 6 = Risk communication | 7 = Surveillance
theme2	Improvement theme discussed and/or referenced	1 = Coordination | 2 = Health systems strengthening | 3 = Human resources | 4 = Preparedness | 5 = Resourcing | 6 = Risk communication | 7 = Surveillance

theme3	Improvement theme discussed and/or referenced	1 = Coordination | 2 = Health systems strengthening | 3 = Human resources | 4 = Preparedness | 5 = Resourcing | 6 = Risk communication | 7 = Surveillance

city	time1	time2	time3	time4	time5	time6	time7	time8	time9	time10	time11	time12	time13	time14	time15	time16
Accra	3	3	3	3	3	3	1	1	1	3	1	1	3	3	3	3
Addis Ababa	2	4	4	3	3	3	3	1	1	1	1	3	3	3	1	5
Amman	2	3	2	2	2	2	2	2	2	2	2	2	2	3	3	3
Athens	2	3	3	3	3	3	5	5	3	3	2	2	3	5	5	5
Bandung	3	3	3	3	3	3	3	3	3	3	3	3	3	3	3	3
Bangkok	3	3	3	2	3	3	2	2	3	3	3	3	3	3	3	3
Barcelona	1	4	4	2	4	4	1	1	1	4	1	1	4	1	1	1
Bengaluru	3	3	3	3	3	3	3	3	3	3	3	3	2	3	3	5
Buenos Aires	1	3	3	3	3	3	3	1	1	2	2	2	1	3	3	3
Cali	1	3	3	3	3	3	3	3	3	3	3	3	3	3	3	3
Colombo	2	2	1	1	1	2	1	1	1	1	1	1	1	1	1	2
Guadalajara	2	2	2	2	2	2	2	2	2	3	3	3	3	3	3	3
Kampala	3	3	3	2	2	3	1	1	1	3	1	3	3	3	3	3
Kigali	1	3	3	1	3	3	3	3	1	1	1	3	3	3	3	3
Kumasi	3	3	3	3	3	3	2	2	3	3	2	3	3	3	3	3
Lima	3	3	3	3	3	3	3	3	3	3	3	3	3	3	3	3
London	5	5	5	3	5	5	1	1	5	5	1	1	5	5	5	5
Lusaka	1	3	3	1	3	3	1	1	1	3	1	1	1	3	3	3
Medellin	1	1	1	2	2	2	1	1	1	1	1	2	1	2	2	2
Ouagadougou	2	3	3	3	3	3	3	3	3	3	3	3	3	3	3	3
Rio de Janeiro	2	3	3	3	3	3	3	3	3	3	3	3	3	3	3	3
Santiago	1	2	2	2	2	2	1	1	1	2	1	2	2	2	2	2
Santo Domingo	3	3	3	3	3	3	3	3	3	3	3	3	3	3	3	3
Vancouver	5	5	5	5	5	5	5	5	5	5	5	5	5	5	5	5
Yangon	2	3	3	3	3	3	3	3	3	3	3	3	3	3	3	3

city	chal1	chal2	chal3	chal4	chal5	chal6	chal7	chal8	chal9	chal10	chal11	chal12	chal13	chal14	chal15	chal16
Accra	1	1	3	1	2	2	3	3	1	3	3	3	2	1	1	1
Addis Ababa	2	6	6	3	4	3	4	2	3	2	2	3	4	3	2	6
Amman	1	1	1	1	1	1	1	1	1	1	1	1	1	1	1	1
Athens	2	1	1	1	1	1	6	6	5	5	2	2	2	6	6	6
Bandung	2	2	2	1	3	2	1	1	2	2	2	3	2	2	2	2
Bangkok	5	1	1	4	3	2	6	5	4	3	2	2	3	2	4	3
Barcelona	3	6	6	3	6	6	3	3	3	6	1	1	6	3	3	3
Bengaluru	2	1	2	1	5	5	4	3	1	1	2	3	2	2	1	6
Buenos Aires	2	1	1	1	1	2	2	3	1	1	3	3	3	2	2	2
Cali	2	2	2	2	2	2	3	3	2	2	3	3	2	2	2	2
Colombo	3	5	5	5	5	3	5	3	2	2	5	3	4	3	4	3
Guadalajara	3	2	2	1	3	3	5	5	3	3	4	2	3	3	3	2
Kampala	2	1	2	2	3	2	3	3	4	4	3	3	3	3	4	3
Kigali	2	1	1	1	1	1	5	5	5	5	5	5	5	5	5	4
Kumasi	1	1	1	1	5	2	2	3	2	1	5	5	3	2	1	3
Lima	2	1	1	2	2	2	3	2	2	2	2	2	2	2	2	2
London	6	6	6	1	6	6	3	2	6	6	3	3	6	6	6	6
Lusaka	2	1	6	2	4	1	4	5	4	3	5	4	1	4	1	1
Medellin	2	2	2	2	2	2	2	2	2	2	2	2	2	2	2	2
Ouagadougou	2	4	4	3	4	4	5	5	4	4	3	4	6	3	2	4
Rio de Janeiro	1	1	1	1	3	3	4	4	5	4	4	4	2	1	1	2
Santiago	2	2	2	1	2	1	3	3	2	2	3	3	3	3	2	2
Santo Domingo	.	.	.	.	.	.	.	.	.	.	.	.	.	.	.	.
Vancouver	6	6	6	6	6	6	6	6	6	6	6	6	6	6	6	6
Yangon	1	2	2	2	2	2	2	2	1	1	1	1	1	1	1	1

city	cchal1	cchal2	cchal3	cchal4	cchal5	cchal6	cchal7	supp1	supp2	supp3	supp4	involve
Accra	3	3	3	3	3	3	3	1	1	1	1	2
Addis Ababa	1	4	4	4	2	3	2	1	1	1	1	2
Amman	1	1	1	1	1	1	1	1	0	0	1	2
Athens	2	2	2	2	2	1	2	1	0	0	1	2
Bandung	3	3	3	3	4	3	2	1	1	1	1	2
Bangkok	3	2	1	2	2	2	1	1	1	1	1	2
Barcelona	2	3	3	2	3	3	2	1	1	1	1	2
Bengaluru	4	5	4	4	5	4	2	0	1	1	1	2
Buenos Aires	2	2	2	2	3	3	2	1	1	1	0	2
Cali	3	3	3	3	3	2	2	1	1	1	1	2
Colombo	5	3	3	3	3	3	3	1	1	1	1	2
Guadalajara	4	4	4	3	5	3	2	1	1	1	1	2
Kampala	3	2	3	2	3	2	2	1	0	0	1	2
Kigali	5	5	3	5	5	5	5	0	0	0	1	2
Kumasi	5	4	3	2	5	2	1	1	1	1	1	2
Lima	2	3	3	3	3	1	1	1	1	0	1	2
London	4	3	3	3	4	2	1	0	0	0	1	2
Lusaka	4	4	4	4	5	5	5	1	1	0	1	2
Medellin	3	2	3	1	3	3	1	1	1	1	1	2
Ouagadougou	5	4	5	5	5	4	3	1	0	0	0	2
Rio de Janeiro	1	1	1	1	1	1	1	1	0	0	0	2
Santiago	2	2	2	2	2	2	1	1	1	1	0	2
Santo Domingo	.	.	.	.	.	.	.	.	.	.	.	2
Vancouver	3	3	4	3	3	2	2	1	0	1	1	2
Yangon	1	1	1	1	1	1	1	1	0	0	1	1

**city**	**theme1**	**theme2**	**theme3**
Accra	5	.	.
Addis Ababa	1	2	4
Amman	5	6	.
Athens	.	.	.
Bandung	6	7	.
Bangkok	4	4	7
Barcelona	1	6	7
Bengaluru	2	2	3
Buenos Aires	3	6	7
Cali			
Colombo	2	3	7
Guadalajara	2	4	7
Kampala	6	7	.
Kigali	6	.	.
Kumasi	3	6	6
Lima	1	5	7
London	1	5	7
Lusaka	2	4	5
Medellin	4	6	.
Ouagadougou	6	7	.
Rio de Janeiro	6	.	.
Santiago	1	6	.
Santo Domingo	.	.	.
Vancouver	4	4	.
Yangon	6	6	7
